# Molecular characterization of hypothetical scaffolding-like protein S1 in multienzyme complex produced by *Paenibacillus curdlanolyticus* B-6

**DOI:** 10.1186/s13568-019-0896-0

**Published:** 2019-10-31

**Authors:** Patthra Pason, Junjarus Sermsathanaswadi, Rattiya Waeonukul, Chakrit Tachaapaikoon, Sirilak Baramee, Khanok Ratanakhanokchai, Akihiko Kosugi

**Affiliations:** 1 Pilot Plant Development and Training Institute (PDTI), King Mongkut’s University of Technology Thonburi (KMUTT), Bangkok, 10150 Thailand; 2 Department of Chemical Technology, Faculty of Science and Technology, Suan Dusit University, 295 Rajasrima Road, Dusit, Bangkok, 10300 Thailand; 3 School of Bioresources and Technology, King Mongkut’s University of Technology Thonburi (KMUTT), Bangkuntien, Bangkok, 10150 Thailand; 4 Biological Resources and Post-harvest Division, Japan International Research Center for Agricultural Sciences (JIRCAS), 1-1 Ohwashi, Tsukuba, Ibaraki 305-8686 Japan

**Keywords:** *Paenibacillus curdlanolyticus*, Hypothetical protein, Surface layer homology domain, Xylanase, Multienzyme complex, Carbohydrate-binding domain

## Abstract

*Paenibacillus curdlanolyticus* B-6 produces an extracellular multienzyme complex containing a hypothetical scaffolding-like protein and several xylanases and cellulases. The largest (280-kDa) component protein, called S1, has cellulose-binding ability and xylanase activity, thus was considered to function like the scaffolding proteins found in cellulosomes. S1 consists of 863 amino acid residues with predicted molecular mass 91,029 Da and includes two N-terminal surface layer homology (SLH) domains, but most of its sequence shows no homology with proteins of known function. Native S1 (nS1) was highly glycosylated. Purified nS1 and recombinant Xyn11A (rXyn11A) as a major xylanase subunit could assemble in a complex, but recombinant S1 (rS1) could not interact with rXyn11A, indicating that S1 glycosylation is necessary for assembly of the multienzyme complex. nS1 and rS1 showed weak, typical endo-xylanase activity, even though they have no homology with known glycosyl hydrolase family enzymes. S1 and its SLH domains bound tightly to the peptide-glycan layer of *P. curdlanolyticus* B-6, microcrystalline cellulose, and insoluble xylan, indicating that the SLHs of S1 bind to carbohydrate polymers and the cell surface. When nS1 and rXyn11A were co-incubated with birchwood xylan, the degradation ability was synergistically increased compared with that for each protein; however synergy was not observed for rS1 and rXynA. These results indicate that S1 may have a scaffolding protein-like function by interaction with enzyme subunits and polysaccharides through its glycosylated sites and SLH domains.

## Introduction

Plant biomass, which has potential as a renewable resource, contains a complex mixture of polysaccharides, such as cellulose, hemicellulose (xylan and galactomannan), pectic substances (polysaccharides comprising mainly 1,4-linked α-d-galactosyluronic acid such as galacturonan), other polysaccharides (e.g., fuco-xyloglucan) (Caffall and Mohnen 2009), and lignin, which is a complex polymer of phenylpropane units. The efficient hydrolysis of these polysaccharides requires not only β-1,4-glycosidic chain-cleaving enzymes, such as endo-β-1,4-glucanase and β-glycosidase, but also the cooperation of other enzymes such as β-1,4-xylanases and side chain-cleaving enzymes like α-l-arabinofuranosidase (Caffall and Mohnen 2009). The xylan-degrading enzymes have potential industrial and commercial applications in areas such as food engineering using xylooligosaccharides and cellulose pulping (Pauly and Keegstra 2010; Saha 2003).

A facultative anaerobic bacterium *Paenibacillus curdlanolyticus* B-6, isolated from an anaerobic digester fed pineapple waste, produces numerous cellulolytic/xylanolytic enzymes (Pason et al. 2006). *P. curdlanolyticus* B-6 also produces a cellulosome-like unique multienzyme complex system of at least 11 protein subunits associated in a 1450-kDa complex by distinct cohesin–dockerin interactions (Pason et al. 2006). This extracellular complex is composed of a 280-kDa scaffolding-like core protein (S1), several minor xylanases and cellulases, and major xylanases of ~ 40 kDa (Pason et al. 2010). Protein S1 of the multienzyme complex was isolated through four chromatographic steps and has a xylan-degrading ability (Pason et al. 2010).

S1 may assemble the multienzyme complex using a mechanism distinct from the cohesin–dockerin interactions of the cellulosome (Bayer et al. 2004, 2008; Demain et al. 2005; Doi and Kosugi 2004; Lynd et al. 2002), because the major xylanase subunit (Xyn11A) of the multienzyme complex does not have a dockerin domain-like structure that is necessary to bind the cohesin domains present in cellulosome scaffolding proteins (Pason et al. 2010; Sermsathanaswadi et al. 2014). In typical cellulosome-producing, anaerobic, cellulolytic bacteria (e.g., *Hungateiclostridium thermocellum*), the cellulosome (2.0–3.5 MDa) consists of a large (197 kDa), non-catalytic, multimodular scaffolding protein CipA, which includes nine cohesins, four hydrophilic modules, and a family 3 carbohydrate-binding module (CBM3). The enzymatic units are noncovalently assembled to the scaffolding protein through high-affinity type I interactions between the dockerin domains of the catalytic units and cohesins on the scaffold. S1 has a large molecular mass and a cellulose binding ability, and it forms a multienzyme complex, which is similar to that of known scaffolding proteins. Consequently, it would be interesting to determine whether the S1 protein acts as a scaffolding protein at the molecular level. In this study, we report the molecular characterization of the scaffolding-like protein S1 from a multienzyme complex produced by *P. curdlanolyticus* B-6.

## Materials and methods

### Bacterial strains and culture medium

*Paenibacillus curdlanolyticus* B-6 is in the Culture Collection of the National Center for Genetic Engineering and Biotechnology, Thailand (accession number BCC 11175). Strain B-6 was grown on Bergey’s mineral salt medium (pH 7.0) supplemented with birchwood xylan (Sigma-Aldrich, St. Louis, MO, USA) as previously reported (Pason et al. 2006, 2010). *E. coli* JM109 (TaKaRa Bio, Shiga, Japan), *E. coli* Rosetta™ 2 (DE3), *E. coli* BL21 (DE3) (Merck KGaA, Darmstadt, Germany), pET19b, and pET22b (Merck KGaA) served as cloning and expression hosts and protein expression vectors.

### Purification of nS1 protein from culture supernatant

The multienzyme complex, including S1 protein, was purified from the cellulose-binding fraction of *P. curdlanolyticus* B-6 culture supernatant, as previously reported (Pason et al. 2006, 2010). To separate nS1 from the enzyme subunits, the purified multienzyme complex was incubated at 4 °C for 15 h in 1 M sucrose and 5 mM ethylenediaminetetraacetic acid. The nS1 was successfully isolated from the enzymatic subunits using Sephacryl S-500 HR gel filtration chromatography (GE Healthcare, Buckinghamshire, UK). The purity and molecular mass of nS1 (280 kDa) were confirmed by SDS-PAGE (ATTO, Tokyo, Japan), and the protein was concentrated using Amicon Ultra centrifugal filters (Merck Millipore Corp., Darmstadt, Germany).

### Isolation and DNA sequence analysis of S1

To clone the gene encoding S1, N-terminal and internal amino acid sequences were determined from purified S1 protein. For N-terminal amino acid sequencing, the S1 protein was subjected to SDS-PAGE and transferred onto a polyvinylidene difluoride membrane (Merck Millipore Corp.) using a Mini Trans-Blot^®^ electrophoretic transfer cell (Bio-Rad Laboratories, Hercules, CA, USA). The protein bands were determined by staining with 0.1% Ponceau Red (Wako Pure Chemical, Osaka, Japan), and the protein on the membrane was sequenced using the Edman method (Thermo Fisher Scientific, MA, USA). For the internal amino acid sequencing of the S1 protein, the single 280-kDa protein band was extracted from a polyacrylamide gel and digested with trypsin.

To clone the *S1* gene, mixed oligonucleotide primers were designed based on the deduced N-terminal and internal amino acid sequences of S1 (Additional file 1). PCR was carried out in standard conditions for Ex *Taq* polymerase (TaKaRa Bio) according to the manufacturer’s instructions. To isolate the full-length gene encoding S1, genomic-walking PCR was performed using a GenomeWalker Kit (TaKaRa Bio) according to the manufacturer’s instructions. Nucleotide and amino acid sequences were analyzed using the BLASTn and BLASTp programs, respectively (https://blast.ncbi.nlm.nih.gov/Blast.cgi). The Carbohydrate Active Enzymes (CAZY) server (http://afmb.cnrs-mrs.fr/pedro/CAZY) was used for determining glycosyl hydrolase and CBM families. The GenBank accession number for *S1* from *P. curdlanolyticus* B-6 is KP723173.

### Purification of rS1, major xylanase subunit Xyn11A and its derivatives, and glycosylation detection

**Fig. 1:**
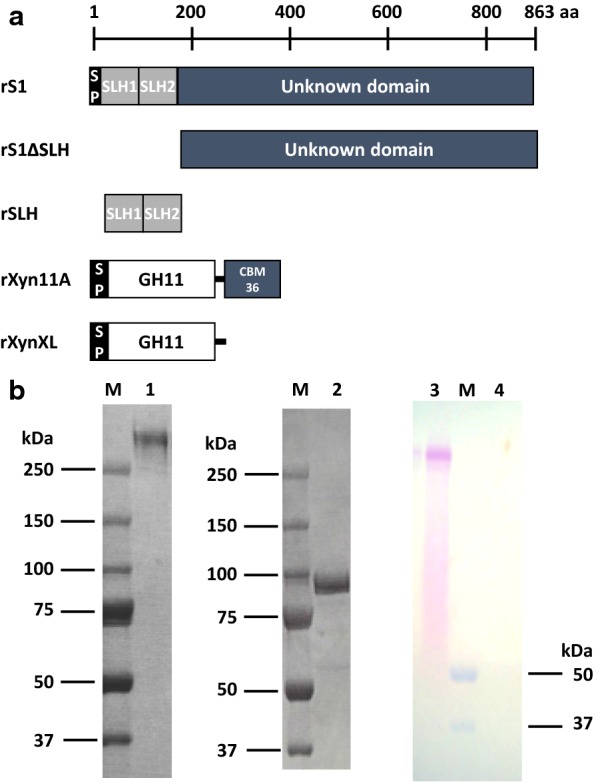
Molecular architecture of *P. curdlanolyticus* B-6 S1 protein (**a**) and the detection of glycoproteins (**b**). Recombinant S1 (rS1), rS1 lacking SLH domains (rS1ΔSLH), recombinant SLH domains (rSLH), recombinant Xyn11A (rXyn11A), and rXyn11A lacking CBM36 domain (rXynXL) were designed and expressed as recombinant proteins in *Escherichia coli* (**a**). SP predicted signal peptide, *aa* number of amino acid sequence. Coomassie Brilliant Blue staining of purified native S1 (nS1) from a partially-purified multienzyme complex (**b**, lane 1) and of rS1 (**b**, lane 2). Glycoprotein staining of nS1 (**b**, lane 3) and rS1 (**b**, lane 4). Lane M, molecular mass markers (kDa). The gel picture does not contain the low molecular weights

The oligonucleotide primers used in this research are listed in Additional file 1. To produce the recombinant proteins rS1 and rSLH (Fig. 1) in *E. coli*, expression plasmids pET-S1 and pET-SLH were prepared using the *S1* gene from *P. curdlanolyticus* B-6. Each recombinant protein was purified with the Profinia Affinity Chromatography Protein Purification System (Bio-Rad Laboratories). Protein concentrations were determined using a Pierce BCA assay kit (Thermo Fisher Scientific, MA, USA) with bovine serum albumin as the standard. Glycoproteins were visualized by staining with a Glycoprotein Staining Kit (G-Biosciences, St. Louis, MO, USA) according to the manufacturer’s instructions. After the initial staining and imaging of glycosylated proteins, the gel was counterstained with RAPID stain solution from the same kit to visualize all the proteins.

### Preparation of cell wall fragments of *P. curdlanolyticus* B-6

*Paenibacillus curdlanolyticus* B-6 cells from 500 ml of a mid-exponential-phase culture were harvested, washed twice with 100 ml of 50 mM phosphate buffer (pH 7.0), and resuspended in 10 ml of the same buffer. The cell suspension was disrupted by sonication, intact cells were removed by centrifuging twice at 1940×*g* for 5 min, and the suspensions were recentrifuged at 39,200×*g* for 20 min. The pellet was suspended in 5 ml of 50 mM phosphate buffer (pH 7.0), treated with 1% SDS by boiling in a water bath for 20 min, and centrifuged at 16,000×*g* for 20 min at room temperature. The supernatant consisted of the cell wall-associated proteins. The pellet, consisting of the cell wall, was resuspended in 5 ml of 50 mM phosphate buffer (pH 7.0) after being washed three times with the same buffer.

### Binding properties of rS1 toward polysaccharides and cell wall fragments

The rS1 protein (50–150 µg) was incubated for 4 h at 30 °C in 200 µl of 50 mM sodium phosphate buffer (pH 7.0) with the prepared cell wall fragments (50–200 µg), microcrystalline cellulose (Sigmacell type 20, Sigma-Aldrich), acid swollen cellulose, insoluble xylan from birchwood, arabinan (Sigma-Aldrich), or chitin (Sigma-Aldrich) with each polysaccharide at a concentration of 10 mg/ml. The reaction mixtures were incubated for 4 h at 30 °C with gentle shaking. The bound and free proteins were separated by centrifugation at 16,000×*g* for 10 min at room temperature. The supernatant consisted of the free protein fraction. A wash fraction was obtained after washing the pellet with 50 mM phosphate buffer (pH 7.0). The pellet, consisting of the insoluble cell wall fragments and attached proteins, was washed with the same buffer and then resuspended in 300 μl of the phosphate buffer. Each fraction was analyzed by SDS-PAGE. To calculate the binding parameters for insoluble carbohydrates, the protein concentrations in the total pellet suspension and free protein were determined using a BCA protein assay kit (Thermo Fisher Scientific). The amount of bound polypeptide was calculated by subtracting the amount of free protein from the amount remaining in the pellet suspension. Affinity (*K*_d_) and binding capacity ([*PC*]max) parameters were calculated using double-reciprocal plots with different fixed levels of bound proteins and insoluble materials, as described previously (Pason et al. 2006).

### In vitro reconstruction of the complex using nS1

In vitro reconstruction was carried out using rXyn11A (Pason et al. 2010), rXynXL (Sermsathanaswadi et al. 2014), rS1, and nS1 purified from the *P. curdlanolyticus* B-6 complex. Purified S1 (approximately 140 μg protein; 0.5 µM) was mixed with rXyn11A (approximately 20 μg protein; 0.5 µM) or rXynXL (approximately 11 μg protein; 0.5 µM) in phosphate-buffered saline containing 1 mM CaCl_2_. To compare differences in their interaction abilities, rS1 (approximately 46 μg protein; 0.5 µM) was also mixed with rXyn11A (approximately 20 μg protein; 0.5 µM) in phosphate-buffered saline buffer containing CaCl_2_. MilliQ water (Millipore Corporation, Billerica, MA, USA) was added to a final volume of 100 μl. The proteins were incubated at room temperature overnight to allow complex formation. A gel-shift experiment with a non-denaturing PAGE analysis was performed. Non-denaturing sample buffer (192 mM glycine, 25 mM Tris; Thermo Fisher Scientific) was added, and 40 μl sample/lane was analyzed by PAGE using a NativePAGE™ Novex^®^ Bis–Tris gel system according to the manufacturer’s instructions (Thermo Fisher Scientific). The cathode buffer contained 1.5 mM Bis–Tris, 5.0 mM tricine, and 0.002% Coomassie Brilliant Blue R-250 (w/v; pH 7.0) (Bio-Rad Laboratories), and the anode buffer contained 5.0 mM Bis–Tris (pH 7.0).

### Enzymatic characterization of S1 protein

Xylanase activity was measured by determining the amount of reducing sugar released from birchwood xylan, oat-spelt xylan, and arabinoxylan (wheat flour) (Sigma-Aldrich) for 15 min incubation (Okada and Shinmyo 1988). Released reducing sugars were quantified using the Somogyi-Nelson method with xylose as a standard (Wood and Bhat 1988). One unit of xylanase activity was defined as the amount of enzyme that liberated 1 μmol of reducing sugar in 1 min under the above conditions. *K*_m_, *V*_max_ and *K*_*cat*_ values for rS1 were determined using the Lineweaver–Burk method (Sermsathanaswadi et al. 2017).

### Hydrolysis of xylooligosaccharides by rS1

rS1 was incubated in 50 mM phosphate buffer (pH 7.0) containing 1 mM xylooligosaccharides (Megazyme International, Wicklow, Ireland) for 1 h at 60 °C. Mono- and oligo-saccharide contents in the supernatants were measured by high-performance liquid chromatography (Shimadzu Corp., Kyoto, Japan) (Sermsathanaswadi et al. 2017). Xylan hydrolysis products were also determined by thin-layer chromatography (Sermsathanaswadi et al. 2017).

### Synergistic effect between nS1 and rXyn11A

Synergistic effects were investigated by incubating aliquots (50 µl) of the protein samples (0.5 µM in 50 mM sodium phosphate buffer, pH 7.0) with 3 ml of birchwood xylan (5 g/l in 50 mM sodium phosphate buffer, pH 7.0) at 60 °C with shaking at 150 rpm. Aliquots (0.2 ml) were extracted at 0, 1, 2, 3, 5, and 10 h, centrifuged, and examined for soluble reducing sugars using xylose as the standard. The released sugars in supernatants were measured using the Somogyi-Nelson method.

### Homology modeling

The SbsB S-layer protein of *Geobacillus stearothermophilus* PV72p2 (PDB accession number 4aq1) (Baranova et al. 2012) and cell wall-anchoring protein of *P. alvei* (PDB accession number 6cwc for the SLH domains) (Blackler et al. 2018) were used as the templates for the construction of a three-dimensional structural model of *P. curdlanolyticus* B-6 S1 in the SWISS-MODEL protein-modeling server (http://swissmodel.expasy.org/), representing the unknown and SLH domains of S1, respectively.

## Results

### S1 from *P. curdlanolyticus* B-6

To determine whether the scaffolding-like S1 protein has a structure similar to that of the cohesins that are normally observed in cellulosomes from cellulolytic clostridia, the *S1* gene was cloned from *P. curdlanolyticus* B-6. An *S1* gene fragment was amplified by PCR using degenerate primers based on the N-terminal amino acid sequence (AEDAQPSTQD, amino acid residues 25–34) by Edman degradation and an internal amino acid sequence in situ by proteolytic enzymes such as trypsin or staphylococcal V-8 protease (DGSIERGYAG, amino acid residues 804–815) of the protein (GenBank accession number KP723173.1). The 5ʹ- and 3ʹ-flanking regions were amplified by PCR-based genome walking and assembled with the known partial sequence. The full-length *S1* gene contains 2589 bp and encodes a protein of 863 amino acids, with a calculated molecular mass of ~ 91 kDa. The N-terminal region showed homology with the S-layer domain-containing hypothetical protein from *Paenibacillus* sp. 32O-W (50% identity, WP_062490275.1) and *Paenibacillus* sp. UNC496MF (43% identity, SFJ26475.1). However, the hypothetical scaffolding-like S1 protein has a different structure from those of known scaffolding proteins, such as CipA and CbpA in cellulosomes (Bayer et al. 2004; Doi and Kosugi 2004; Lynd et al. 2002). According to a BLAST analysis, the first 125 amino acids of S1 were identified as two surface-layer homology (SLH) domain sequences (Fig. 1a). SLH1 (amino acids 24 to 65) and SLH2 (amino acids 82 to 125) had high homology levels with an S-layer domain protein from *Geobacillus* sp. Y412MC10 (69% identity, ZP_03037712) and *Paenibacillus* sp. oral taxon 786 str. D14 (52% identity, ZP_04851624), respectively. We could find no functional similarities to the amino acid sequences following the SLH domains (i.e., from residues 126 to 763) in any databases, including GenBank, EMBL, and DDBJ.

There were four inconsistencies between our sequence analysis and the protein properties of S1 characterized in previous reports (Pason et al. 2006, 2010), as follows: (1) the theoretical mass of the S1 protein based on the sequence (~ 91 kDa) is quite different from the ~ 280-kDa molecular mass determined by SDS-PAGE of native S1 (nS1); (2) in general, cohesin–dockerin modules are necessary for interactions between a scaffolding protein and enzymatic subunits in known multienzyme complexes such as the cellulosome (Doi and Kosugi 2004), but these modules are not present in the S1 protein sequence. Nevertheless, a multienzyme complex prepared from *P. curdlanolyticus* B-6 is tightly assembled from nS1, major xylanase (Xyn11A), and several minor cellulases and xylanases; (3) when a zymogram analysis of this multienzyme complex was carried out with birchwood xylan as the substrate, the xylan degradation ability was clearly observed at ~ 280 kDa, corresponding to the size of nS1; (4) the enzyme complex of *P. curdlanolyticus* B-6 can bind microcrystalline cellulose even though there is no clear CBM in the S1 protein sequence. To understand these inconsistencies, the nS1 protein, recombinant S1 (rS1) protein, some derivatives, and Xyn11A (Fig. 1a), as the major associated enzyme subunit, were analyzed.

### Different molecular masses of nS1 and rS1

The nS1 protein was partially prepared from a culture supernatant of *P. curdlanolyticus* B-6 using ammonium sulfate precipitation and ion and hydrophobic interaction chromatography (Pason et al. 2010). The molecular masses of the purified nS1 and rS1 from *Escherichia coli* were compared by SDS-PAGE (Fig. 1b). The molecular mass of nS1 was ~ 280 kDa, compared with ~ 91 kDa for rS1. An analysis using a glycoprotein staining kit revealed that this difference in molecular mass resulted from the glycosylation of nS1. rS1 was not glycosylated (Fig. 1b).

### Complex formation with recombinant Xyn11A

To confirm whether the S1 protein can play the role of a scaffolding protein that interacts with enzyme subunits in a multienzyme complex, analogous to the cellulosome, interaction tests were carried out using nS1 or rS1 as the scaffolding protein and rXyn11A. Xyn11A, consisting of a glycosyl hydrolase family (GH) 11 xylanase catalytic domains, a linker sequence, and a family 36 CBM, was characterized as the major xylanase in the multienzyme complex from *P. curdlanolyticus* B-6 (Pason et al. 2010; Sermsathanaswadi et al. 2014). However, Xyn11A does not have any modules, such as dockerin, that are necessary for the interactions of enzymes with scaffolding proteins in the cellulosome.

**Fig. 2:**
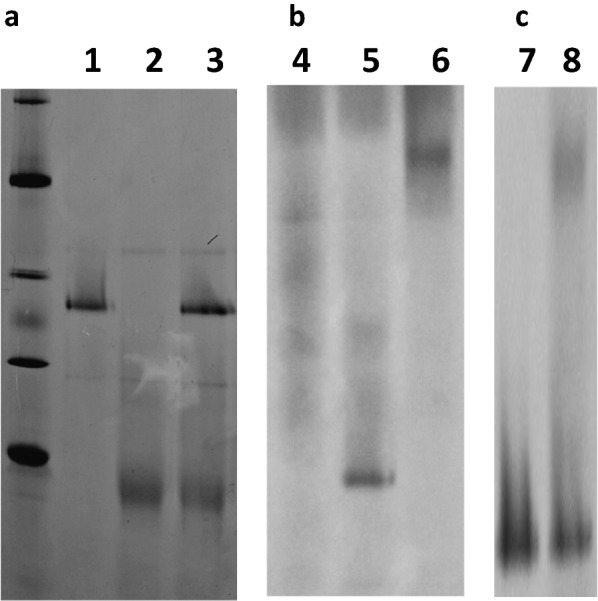
Analysis of enzyme-complex formation by NativePAGE™. Electrophoretic mobility of components and assembled complexes on nondenaturing gels. Recombinant S1 (rS1), native S1 (nS1), rXyn11A and rXynXL (Xyn11A lacking the family 36 carbohydrate-binding module) were allowed to interact overnight at room temperature. Lane 1, rS1; lane 2, rXyn11A; lane 3; interaction of rS1 and rXyn11A; lane 4, nS1; lane 5, rXyn11A; lane 6, interaction of nS1 and rXyn11A; lane 7, rXynXL; lane 8, interaction of nS1 and rXynXL. Lanes 1–3 are from the same nondenaturing gel. Lanes 4–6 and lanes 7–8 were cropped from different nondenaturing gels

A blue native polyacrylamide gel was used to investigate the interactions between S1s and rXyn11A. Incubating rS1 with rXyn11A did not result in any observed interactions (Fig. 2a). In contrast, nS1 and rXyn11A interacted, migrating at a greater molecular mass than each individual protein (Fig. 2b). Thus, the glycosylation of nS1 may be important for the interaction of S1 with Xyn11A, at least in multienzyme complex formation. Thus, to test whether the CBM of rXyn11A bound to glycosyl chains of nS1, nS1 was incubated with rXynXL (Fig. 1a), a form of Xyn11A from which the family 36 CBM was removed (Sermsathanaswadi et al. 2014). nS1 could interact with rXynXL (Fig. 2c), but the interaction appeared weaker than that between nS1 and intact rXyn11A. Thus, CBMs in enzyme subunits might contribute to the assembly of the *P. curdlanolyticus* B-6 multienzyme complex through weak binding to the glycosyl side chains of S1.

### Xylan degradation properties of S1

No GH family catalytic domains are predicted in S1 based on its sequence. Nevertheless, a xylan hydrolysis ability of nS1 was seen when the partially purified multienzyme complex was applied to a zymogram analysis in which birchwood xylan was the substrate (Pason et al. 2010). To determine whether S1 has a xylan degradation ability, the hydrolytic abilities of nS1 and rS1 were characterized for xylan and other substrates having glycosidic bonds. nS1 and rS1 had clear hydrolytic abilities toward soluble and insoluble birchwood xylan, but no activities toward microcrystalline cellulose, CMC, β-glucan, starch, arabinan, xyloglucan, chitin, or *p*-nitrophenol substrates (Additional file 2). The xylan degradation activities of nS1 were 0.69 ± 0.05 and 0.38 ± 0.04 U/mg protein toward soluble and insoluble birchwood xylan, respectively, while the activities of rS1 were 0.55 ± 0.02 and 0.36 ± 0.01 U/mg protein, respectively. Thus, the glycosylation of S1 may not influence the xylan degradation ability.

**Fig. 3:**
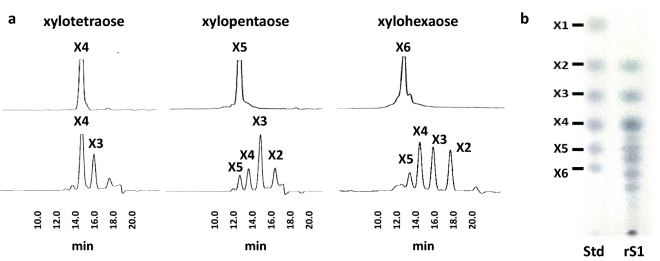
End-product analysis of xylooligosaccharides and xylan hydrolyzed by rS1. The end products were analyzed by high-performance liquid chromatography (**a**). Thin-layer chromatography of hydrolysis products of soluble birchwood xylan (**b**). Authentic xylooligosaccharides were used as standards (lane Std). X1, xylose; X2 to X6, xylobiose to xylohexaose

The xylanase activity of nS1 decreased by 80% with the addition of 1 mM ethylenediaminetetraacetic acid, and the presence of divalent cations (1 mM Ca^2+^ or Mg^2+^) enhanced the xylanase activity, indicating that the protein may contain a Ca^2+^-binding site that is functionally important (Additional file 3). Kinetic parameters were determined for nS1 and rS1 using soluble birchwood xylan as the substrate (Table 1). rXyn11A has high *K*_*m*_ and *K*_*cat*_ (Sermsathanaswadi et al. 2014) values compared with those of S1. nS1 showed lower kinetic parameters than that of rS1 (Table 1), however, similar optimum reaction pH levels (pH 6.0) and temperatures (60 °C), for xylan degradation. When rS1 was incubated with xylooligosaccharides, such as xylotetraose, xylopentaose, and xylohexaose, and birchwood xylan, the major products were xylotetraose and xylotriose; however, nS1 and rS1 cannot degrade to xylose (Fig. 3a, b).

**Table 1:** Enzymatic characterization of nS1, rS1 and rXyn11A

Property	nS1	rS1	rXyn11A
Molecular mass (kDa)	280	91	40
Structure	SLH-unknown glycoprotein	SLH-unknown	GH11-linker-CBM36
Temperature optimum (stability)	60 °C (< 60 °C)	60 °C (< 60 °C)	60 °C
pH optimum (stability)	6.0 (6.0–8.0)	6.0 (6.0–8.0)	6.0 (6.0–7.0)
*K*_*m*_ (mg/ml)	29.5 ± 2.0	19.5 ± 3.0	1.5 ± 0.1
*K*_*cat*_ (1/s)	66.1 ± 0.1	162.2 ± 2.0	204.4 ± 3.0

Soluble birchwood xylan was used as substrate

Data are presented as the means from three independent experiments. Errors represent standard deviations (n = 3)

*SLH* surface layer homology domain, *GH11* glycosyl hydrolase family 11, *CBM36* family 36 carbohydrate-binding module

### Carbohydrate binding ability of S1

**Fig. 4:**
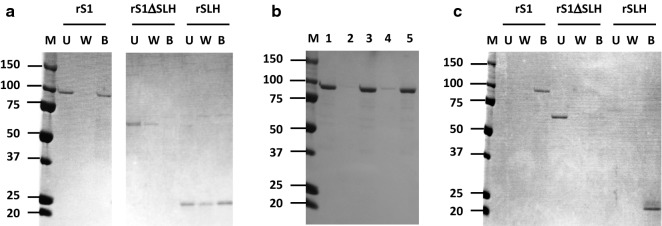
Binding properties of rS1 and its truncated derivatives. Binding to microcrystalline cellulose (**a**), other polysaccharides, such as insoluble xylan and chitin (**b**), and the bacterial peptidoglycan layer (**c**), as revealed by SDS-PAGE. Each recombinant protein was incubated with microcrystalline cellulose, another polysaccharide or the peptidoglycan layer for 4 h at 30 °C. Unbound (lane U) and bound (lane B) protein fractions were separated by centrifugation; pellets were washed three times (lane W). **b** Lane 1, binding fraction of microcrystalline cellulose; lane 2, binding fraction of acid swollen cellulose; lane 3, binding fraction of insoluble birchwood xylan; lane 4, binding fraction of arabinan; lane 5, binding fraction of chitin. Lane M, molecular mass markers (kDa). Lanes U, W, and B of rS1 (**a**) are from an SDS-PAGE gel. Lanes U, W and B of rS1ΔSLH and rSLH were cropped from different SDS-PAGE gels, respectively

The multienzyme complex was prepared from a culture supernatant of *P. curdlanolyticus* B-6 using a combination of cellulose-binding affinity and several column chromatographic steps (Pason et al. 2006, 2010). To determine whether the S1 protein could bind to cellulose and other polysaccharides, binding properties and affinities were characterized using rS1. rS1 could bind to microcrystalline cellulose (Fig. 4a), insoluble xylan, and chitin, but not to acid swollen cellulose or arabinan (Fig. 4b). To determine which regions of the protein are responsible for carbohydrate binding, binding experiments with microcrystalline cellulose were carried out using recombinant S1 with the SLH1 and 2 domains removed (rS1ΔSLH) and with the SLH1 and 2 domains of S1 only (rSLH). When rS1ΔSLH and rSLH were respectively incubated with microcrystalline cellulose, rS1ΔSLH lost the cellulose-binding ability, while rSLH could bind to cellulose (Fig. 4a), indicating that the SLH domains are responsible for binding to cellulose.

The SLH domain functions in anchoring to the cell surface of Gram-positive bacteria by binding to the peptidoglycan layer (PGL) (Blackler et al. 2018; Sára and Sleytr 2000). To determine whether the SLH domains of S1 can also play an anchoring role to this cell surface layer, binding tests were carried out using a PGL prepared from *P. curdlanolyticus* B-6. Although rS1 and rSLH could bind to the PGL, rS1ΔSLH remained in the unbound fraction. Thus, the SLH domains of S1 are bifunctional, binding to the cell surface of *P. curdlanolyticus* B-6 and to microcrystalline cellulose (Fig. 4c). To compare binding properties, the binding affinities of the SLH domains were measured with the PGL, cellulose, and insoluble xylan. rS1 showed a greater binding affinity and lower capacity for the PGL (0.10 ± 0.01 µM and 0.19 ± 0.01 µmol/g, respectively) than for cellulose (0.23 ± 0.02 µM and 0.27 ± 0.01 µmol/g, respectively) or insoluble xylan (0.22 ± 0.01 µM and 0.24 ± 0.01 µmol/g, respectively) (Table 2). rS1ΔSLH lost the ability to bind the PGL and microcrystalline cellulose, but could still bind insoluble xylan (Table 2). Thus, the SLH domains appear to mainly act in cell surface anchoring, while the multienzyme complex involving S1 dissociated from the cell surface may bind to substrates, such as cellulose and xylan, through the SLH domains.

**Table 2:** Binding affinities and capacities of rS1 and its truncated derivatives toward the peptidoglycan cell surface layer and insoluble substrates

Substrates	*K*_d_ (µM)^a^			[*PC*]max (µmol/g substrate)^a^		
	rS1	rS1ΔSLH	rSLH	rS1	rS1ΔSLH	rSLH
Peptidoglycan layer	0.10 ± 0.05	ND^b^	0.12 ± 0.04	0.19 ± 0.1	ND	0.15 ± 0.1
Microcrystalline cellulose	0.23 ± 0.05	ND	0.27 ± 0.05	0.27 ± 0.1	ND	0.23 ± 0.1
Insoluble xylan	0.22 ± 0.04	1.32 ± 0.2	0.32 ± 0.03	0.24 ± 0.1	0.20 ± 0.04	0.20 ± 0.1

Data are presented as the means from three independent experiments. Error bars represent the standard deviation (n = 3)

^a^The binding assay was performed in triplicate, and the *K*_d_ and [*PC*]max values were calculated by using a double-reciprocal plot

^b^ND, no binding detected

### Synergistic xylan degradation of enzyme complex containing S1 and rXyn11A

**Fig. 5:**
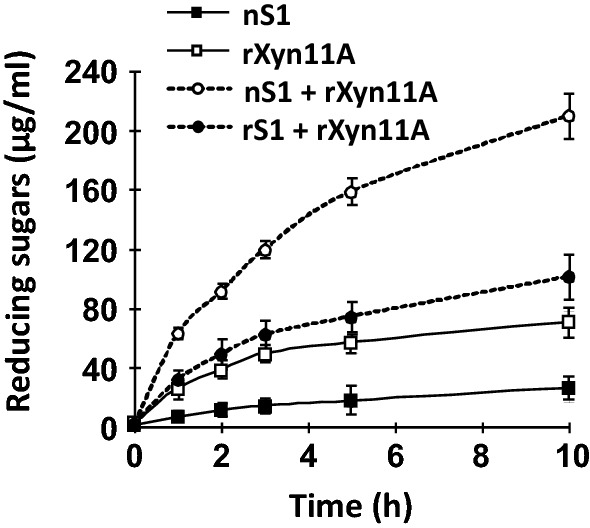
Hydrolysis activity profiles. The enzymatic activities of nS1, rXyn11A, the nS1 plus rXyn11A complex, and the rS1 plus rXyn11A complex toward birchwood xylan were independently determined by measuring the amount of reducing sugar released from 0.5% substrate at 60 °C. The complexes were assembled by mixing nS1 or rS1 with rXyn11A at fixed concentrations (0.5 μM). Data are presented as the means from three independent experiments. Error bars represent the standard deviations (n = 3)

Cellulosome components (the scaffolding protein and enzyme subunits) show synergistic degradation effects when associated with the substrate (Bayer et al. 2004, 2008). To test whether S1 is also able to cooperate in a complex with synergistic effects, experiments were performed with nS1 and rXyn11A using insoluble birchwood xylan as the substrate. The xylan-degrading abilities of nS1 (0.5 µM) and rXyn11A (0.5 µM) individually were 27 µg/ml and 71.0 µg/ml released reducing sugars, respectively (Fig. 5). When nS1 and rXyn11A were incubated together with xylan, the degradation ability was enhanced, to 223 µg/ml released reducing sugar. Thus, the complex had a synergistic xylan-degradation effect. However, synergistic effects were not observed for a mixture of rS1 and rXyn11A (Fig. 5). Thus, S1 may play a scaffolding protein-like role and assemble enzyme subunits. In addition, the glycosylation of S1 is important for the assembly of scaffold subunits.

## Discussion

In this study, the roles of S1, a scaffolding-like protein in a multienzyme complex produced by *P. curdlanolyticus* B-6, in enzyme-complex formation and xylan degradation were characterized. We identified four inconsistencies between the sequence analysis of S1 and the protein properties of S1 reported previously (Pason et al. 2010). Based on the sequence analysis, the molecular mass of S1 was predicted to be ~ 91 kDa, compared with an observed value of ~ 280 kDa for nS1. This difference was the result of glycosylation. As a similar example, the scaffolding protein CipA of the *C. thermocellum* cellulosome is also heavily glycosylated by carbohydrate chains based on galactose and glucose (Demain et al. 2005; Mori 1992). Reported prokaryotic glycoproteins are classified into five major types depending upon their localization and include surface layer glycoproteins, membrane associated glycoproteins, cell-surface glycoproteins, secreted glycoproteins, and exo-enzymes (Upreti et al. 2003). The glycosylation of proteins may enhance their hydrophilic properties and aggregation capabilities in the presence of divalent cations (Paul and Wieland 1987), suggesting that protein–protein interactions through cations may be involved the properties. The glycosylation of S1 plays an important role in assembling the multienzyme complex of *P. curdlanolyticus* B-6, because no complex was formed by (unglycosylated) rS1. *N*-linked glycosylation usually occurs at the Asn of Asn-X-Ser/Thr sequences (J Lechner and Wieland 1989; Upreti et al. 2003), and such motifs are located starting at positions 58, 73, 118, 318, 695, 831, and 852 of the S1 amino acid sequence. Prediction software (NetNGlyc 1.0; http://www.cbs.dtu.dk/services/NetNGlyc/) suggested that positions 58, 73, 118, and 318 may be *N*-glycosylated, and three of these positions (58, 73, and 118) are located in the SLH domains. An analysis of the chemical components of nS1 is necessary to help elucidate the mechanism(s) of protein–protein interactions occurring through the glycosyl chains.

**Fig. 6:**
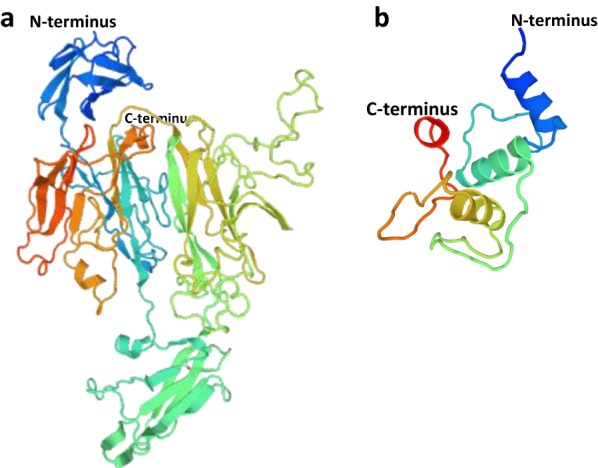
Structural models of *P. curdlanolyticus* B-6 S1 protein. The unknown domain (**a**) and the surface layer homology (SLH1-2) domains (**b**) are shown. Homology models of *P. curdlanolyticus* B-6’s unknown and SLH1–2 domains based on template 4aq1.2 and 6cwc.1, respectively, were predicted using SWISS MODEL

However, the glycosylation of S1 did not appear to play any role in its enzyme activity. We observed that nS1 and rS1 showed similar enzymatic properties, kinetic parameters, and xylan-degradation capacities (Table 1). There are no protein regions with homology to any known GH families, and the 738 amino acids after the SLH domains shows no functional homology with any sequence in any public database. The β-1,4-xylanases are generally included in GH10 and GH11 based on primary structure similarity and three-dimensional structural homology levels. GH11 xylanases are highly specific, displaying exclusive substrate specificity toward xylose-containing substrates and a preference for insoluble polymeric substrates. S1 shows a high substrate specificity toward xylan (Additional file 2); however, the enzymatic affinity and capacity are remarkably low compared with those of rXyn11A (Table 1). The GH11 proteins contain a single major α-helix and two extended pleated β-sheets that form a jelly-roll fold (Paës et al. 2012). The structural features include a compact globular structure and a thumb-like structure as an 11-residue loop that contains the active site (Paës et al. 2012; Wakarchuk et al. 1994). When S1 was analyzed by three-dimensional structural modeling based on the SbsB S-layer protein from *G. stearothermophilus* (PDB code 4aq1.2), as the most similar protein (69.5% amino acid similarity), an immunoglobulin-like fold (Baranova et al. 2012) was observed in the modeled structure (Fig. 6a). The catalytic machinery of GH11 is composed of two glutamate residues, acting as a nucleophile and an acid/base catalyst, respectively, located in the middle of the long cleft (Paës et al. 2012). Although 46 glutamate residues and many β-strand structures were observed in the model of S1, a cleft that spans the entire molecule could not clearly be identified. Thus, additional experiments, such as three-dimensional structure and domain function analysis, are necessary to determine whether S1 contains a novel type of catalytic domain.

SLHs of Gram-positive bacteria generally have covalently bound carbohydrate chains. However, as far as we know, the carbohydrate-binding ability of the SLH domain has not been reported. Typically, S-layer proteins have a high acidic and hydrophobic amino acid contents. The SLH domains of S1 also contained many hydrophobic amino acids, including 1, 2.5, and 4.5 mol% tryptophan, tyrosine, and phenylalanine, respectively. This ratio is similar to that of the novel proposed CBM region of the mature CttA protein (0.78, 3.66, and 2.62 mol% tryptophan, tyrosine, and phenylalanine, respectively) (Rincon et al. 2007). CBMs have been extensively characterized in CAZY database (http://www.cazy.org/) and grouped into three types reflecting both their mode of ligand recognition and the substrate of glycan (Boraston et al. 2004). Type A CBMs bind to the surface of crystalline polysaccharides, type B proteins interact with internal regions of single glycan chains (endo-type), and type C modules recognize the termini of glycan chains (exo-type) (Boraston et al. 2004; Gilbert et al. 2013). Type A CBMs use a flat surface populated with aromatic residues to bind to crystalline cellulose, whereas type B CBMs use a deep groove to bind individual twisted glucan chains found in disordered cellulose (Boraston et al. 2004). A modeling result based on SLH domains from *P. alvei* (SWISS-MODEL: 6cwc.1) (Blackler et al. 2018) showed structures with three helices, quite different from known type A and B CBM structures (Fig. 6b). It is unknown whether the aromatic residues of SLH play an important role in cellulose and xylan binding. Thus, molecular research, such as replacement of the aromatic residues, is necessary to obtain a further understanding of the SLH domains’ affinities for cellulose and xylan.

Efficient cellulose degradation by *C. thermocellum* is essentially dependent on the formation of the cellulosome complex, mediated by the primary scaffoldin protein CipA (Bayer et al. 2004, 2008). Nevertheless, examples of other protein–protein assemblies include a unique mechanism of carbohydrate gluing to yield the complete quaternary supramolecular structure of an annelid giant hemoglobin (Ebina et al. 1995), in which carbohydrate acts noncovalently to glue together the components. In addition, glycosyl hydrolase subunits with CBMs might be able to bind to glycosyl side chains of glycoproteins. It is not clear whether carbohydrate gluing and/or CBM-equipped subunits are involved in the assembly of the *P. curdlanolyticus* B-6 multienzyme complex. However, glycosyl side chains of S1 may be important in the formation of the supramolecular complex because non-glycosylated S1 (i.e., rS1) lost the ability to assemble this complex. When nS1 and rXyn11A were incubated with xylan, synergistic xylan-degradation effects were observed. Similarly, the degradation of crystalline cellulose was reportedly increased by adding the scaffolding protein CipA to the cellulosomal subunits (Fierobe et al. 2002; Hirano et al. 2015). Thus, our results indicate that nS1 may have a scaffolding-like function, even though the multienzyme complex produced by *P. curdlanolyticus* B-6 uses a different manner of assembly from that of the cellulosome. According to a BLAST analysis, S1-like proteins containing S-layer domains are found in several *Paenibacillus* species, such as *P. beijingensis*, and *Paenibacillus* sp. 32O-W, even though they share low amino acid sequence identities (40–42%). This is the first report to functionally characterize the hypothetical surface layer-protein S1. A further characterization of S1 may reveal novel protein–protein interactions and a novel GH family.

## Supplementary information

**Additional file 1.** Oligonucleotide primers used for S1 gene cloning and construction of rS1 and its truncated derivatives.

**Additional file 2.** Substrate specificity of nS1 and rS1.

**Additional file 3.** Effects of cations and chelating reagents on the xylanase activity of nS1.

## Data Availability

The datasets used and/or analyzed for the current study are available from the corresponding author upon reasonable request.

## Abbreviations

CMC: carboxymethyl cellulose
SLH: surface layer homology
rSLH: recombinant SLH
nS1: native S1
rS1: recombinant S1
rXyn11A: recombinant Xyn11A
CBM: carbohydrate-binding module
SDS-PAGE: sodium dodecyl sulfate-polyacrylamide gel electrophoresis
GH: glycosyl hydrolase family
PGL: peptidoglycan layer
