# Supplementary material for: Molecular characterization of hypothetical scaffolding-like protein S1 in multienzyme complex produced by Paenibacillus curdlanolyticus B-6
Source: AMB Express. 2019 Oct 31;9:171. doi: 10.1186/s13568-019-0896-0 (PMC6823336; doi:10.1186/s13568-019-0896-0)
Supplement: Supplementary file 1 — Additional file 1. Oligonucleotide primers used for S1 gene cloning and construction of rS1 and its truncated derivatives. [file 13568_2019_896_MOESM1_ESM.docx]

Additional file 1. Oligonucleotide primers used for S1 gene cloning and construction of rS1 and its truncated derivatives.

| Cloning/Protein | Orientation^a^ | Sequence (5′→3′) |
| --- | --- | --- |
| S1 (N-terminal sequence) | F | GCNGARGAYGCNGARCCNWSNACNGARGAY |
| S1 (internal sequence) | R | ARNCCNGCNGGYTCDATNACNACNCCRTAYTGRTC |
| rS1 | F | GGAATTCCATATGGCTGAAGACGCACAACCGAG |
|  | R | CCGCTCGAGAGCTGCATCGGAAGCCGTAAC |
| rS1∆SLH^b^ | F | CATGCCATGGCTGCTCAAACGGGCGCTAA |
| rSLH^b^ | R | CCGCTCGAGTTAGCGCCCGTTTGAGCAGC |
| rXyn11A | F | GGAATTCCATATGGTA ACGATTACGAAT |
|  | R | ATTGCTCAGCATTGATTTCCAAATAATCGA |
| rXynXL^b^ | R | ATTGCTCAGCACCTGTATTACCGCCGCCA |

^a^ F, forward; R, reverse.

^b^ The reverse primer for rS1 and forward primers for rS1 and rXyn11A were used for construction of rS1∆SLH, rSLH, and rXynXL, respectively.
