# Supplementary material for: Molecular characterization of hypothetical scaffolding-like protein S1 in multienzyme complex produced by Paenibacillus curdlanolyticus B-6
Source: AMB Express. 2019 Oct 31;9:171. doi: 10.1186/s13568-019-0896-0 (PMC6823336; doi:10.1186/s13568-019-0896-0)
Supplement: Supplementary file 2 — Additional file 2. Substrate specificity of nS1 and rS1. [file 13568_2019_896_MOESM2_ESM.docx]

Additional file 2. Substrate specificity of nS1 and rS1

| Substrate | Specific activity (U/mg protein)^a^ | |
| --- | --- | --- |
|  | nS1 | rS1 |
| Soluble birchwood xylan | 0.69 ± 0.05 | 0.55 ± 0.02 |
| Insoluble birchwood xylan | 0.38 ± 0.04 | 0.36 ± 0.01 |
| Microcrystalline cellulose | nd^b^ | nd |
| Carboxymethyl cellulose | nd | nd |
| Chitin | nd | nd |
| Debranched arabinan | nd | nd |
| Galactomannan | nd | nd |
| Glucomannan (locust bean gum) | nd | nd |
| Barley β-glucan | nd | nd |
| Laminaran | nd | nd |
| Rhamnogalacturonan | nd | nd |
| Starch (soluble) | nd | nd |
| Arabinogalactan from larch wood | nd | nd |
| Xyloglucan | nd | nd |
| ^c^*p*NP-β-D-xylopyranoside | nd | nd |
| *p*NP*-*β-D-glucopyranoside | nd | nd |
| *p*NP-β-D-cellopyranoside | nd | nd |
| *p*NP-β-L-arabinofuranoside | nd | nd |

Glycosyl hydrolase activities were measured based on the amount of reducing sugar liberated from other polysaccharides at 0.5% (v/w) final concentration. β-Glucosidase, β-xylosidase and α-L-arabinofuranosidase activities were determined based on measurement of *p*-nitrophenol (*p*-NP) release from *p*-NP-substrates.

^a^ One unit (U) of enzyme activity was defined as the amount of enzyme that liberated 1 µmol of reducing sugar in 1 min. The substrate concentration used for specific activity determination was 10 mg/ml for all polysaccharide substrates. The released reducing sugars were determined using the Somogyi-Nelson method. Results are means ± standard deviations (n = 3).

^b^ nd: Not activity detected.

^c^ *p*NP, *p*-nitrophenyl.
