# Supplementary material for: Molecular characterization of hypothetical scaffolding-like protein S1 in multienzyme complex produced by Paenibacillus curdlanolyticus B-6
Source: AMB Express. 2019 Oct 31;9:171. doi: 10.1186/s13568-019-0896-0 (PMC6823336; doi:10.1186/s13568-019-0896-0)
Supplement: Supplementary file 3 — Additional file 3. Effects of cations and chelating reagents on the xylanase activity of nS1. [file 13568_2019_896_MOESM3_ESM.docx]

Additional file 3. Effects of cations and chelating reagents on the xylanase activity of nS1

| Compound added^a^ | Activity (%)^b^ |
| --- | --- |
|  | nS1 |
| No addition | 100 |
| EDTA | 17 ± 1 |
| Triton X-100 | 117 ± 2 |
| SDS | 11 ± 1 |
| NaCl | 56 ± 4 |
| KCl | 50 ± 2 |
| CaCl_2_ | 205 ± 4 |
| MgCl_2_ | 167 ± 2 |
| CuSO_4_ | 44 ± 2 |

^a^ The cationic ions or chelating reagents were present at 1-mM final concentrations in reaction mixtures.

^b^ The activity was defined as 100% when no exogenous chemicals were added to the reaction.

Results are means ± standard deviations (n = 3).

EDTA, ethylenediaminetetraacetic acid; SDS, sodium dodecyl sulfate.
